# Clinical Practice Guidelines for the Immunological Management of Chromosome 22q11.2 Deletion Syndrome and Other Defects in Thymic Development

**DOI:** 10.1007/s10875-022-01418-y

**Published:** 2023-01-17

**Authors:** Peter J. Mustillo, Kathleen E. Sullivan, Ivan K. Chinn, Luigi D. Notarangelo, Elie Haddad, E. Graham Davies, Maria Teresa de la Morena, Nicholas Hartog, Joyce E. Yu, Vivian P. Hernandez-Trujillo, Winnie Ip, Jose Franco, Eleonora Gambineri, Scott E. Hickey, Elizabeth Varga, M. Louise Markert

**Affiliations:** 1 Division of Allergy and Immunology, Department of Pediatrics, Nationwide Children’s Hospital, Columbus, OH 43205 USA; 2 Division of Allergy Immunology, Department of Pediatrics, Children’s Hospital of Philadelphia, Philadelphia, PA 19104 USA; 3 Division of Immunology, Allergy, and Retrovirology, Department of Pediatrics, Texas Children’s Hospital, Houston, TX 77030 USA; 4 National Institute of Allergy and Infectious Diseases, National Institutes of Health, Bethesda, MD 20892 USA; 5 Department of Pediatrics, Department of Microbiology, Infectious Diseases and Immunology, CHU Sainte-Justine, University of Montreal, Montreal, QC H3T 1C5 Canada; 6 Department of Immunology, Great Ormond Street Hospital and UCL Great Ormond Street Institute of Child Health, London, WC1N 3HJ UK; 7 Division of Immunology, Department of Pediatrics, Seattle Children’s Hospital, University of Washington, Seattle, WA 98105 USA; 8 Spectrum Health Helen DeVos Children’s Hospital Department of Allergy and Immunology, Michigan State University College of Human Medicine, East Lansing, USA; 9 Division of Allergy, Immunology & Rheumatology, Department of Pediatrics, Columbia University Irving Medical Center, New York, NY USA; 10 Division of Allergy and Immunology, Department of Pediatrics, Nicklaus Children’s Hospital, Miami, FL USA; 11 Department of Immunology, Great Ormond Street Hospital and UCL Great Ormond Street Institute of Child Health, London, WC1N 3JH UK; 12 Grupo de Inmunodeficiencias Primarias, Facultad de Medicina, Universidad de Antioquia UdeA, Medellin, Colombia; 13 Department of “NEUROFARBA”, Section of Child’s Health, University of Florence, Florence, Italy; 14 Centre of Excellence, Division of Pediatric Oncology/Hematology, Meyer Children’s Hospital IRCCS, Florence, Italy; 15 Division of Genetic & Genomic Medicine, Department of Pediatrics, Nationwide Children’s Hospital, Columbus, OH 43205 USA; 16 Institute for Genomic Medicine, Nationwide Children’s Hospital, Columbus, OH 43205 USA; 17 Department of Immunology, Duke University Medical Center, Durham, NC 27710 USA

**Keywords:** 22q11.2 deletion, DiGeorge syndrome, Defects in thymic development, CHARGE syndrome, Immunology guidelines, Thymic implant

## Abstract

Current practices vary widely regarding the immunological work-up and management of patients affected with defects in thymic development (DTD), which include chromosome 22q11.2 microdeletion syndrome (22q11.2del) and other causes of DiGeorge syndrome (DGS) and coloboma, heart defect, atresia choanae, retardation of growth and development, genital hypoplasia, ear anomalies/deafness (CHARGE) syndrome. Practice variations affect the initial and subsequent assessment of immune function, the terminology used to describe the condition and immune status, the accepted criteria for recommending live vaccines, and how often follow-up is needed based on the degree of immune compromise. The lack of consensus and widely varying practices highlight the need to establish updated immunological clinical practice guidelines. These guideline recommendations provide a comprehensive review for immunologists and other clinicians who manage immune aspects of this group of disorders.

## Introduction

Current practices regarding the immunological work-up and management of patients affected with chromosome 22q11.2 microdeletion syndrome (22q11.2del) and other defects in thymic development (DTD) are widely variable. Among these disorders are DiGeorge syndrome (DGS), also termed DiGeorge anomaly, and coloboma, heart defect, atresia choanae, retardation of growth and development, genital hypoplasia, ear anomalies/deafness (CHARGE) syndrome. Clinical practice varies in the initial and subsequent laboratory evaluations, terminology used to describe the condition and one’s immune status, criteria used for administration of live vaccines, and how often follow-up is needed based on the degree of immune compromise. Factors influencing these differences include a provider’s individual training and experience, source(s) of literature reviewed, and access to specific immunological laboratory studies. The lack of consensus and widely varying practices highlights the need to establish updated immunological clinical practice guidelines and make them accessible for any clinician involved in the care of affected individuals.

In late 2020, an immunology workgroup was formed with the specific intent of reviewing and addressing these widely varying approaches and working to offer comprehensive management guidelines. The workgroup consisted of members of the Advocacy Committee of the Clinical Immunology Society with clinical immunologists considered experts in the field. Based on experience related to 22q11.2del/DGS and other DTD, identified experts were invited to participate from five countries including Canada, Colombia, Italy, the UK, and the USA. In total, surveys were sent to 13 invited physicians who agreed to participate, consisting of 39 questions assessing their individual approach related to the diagnosis and immunologic management of patients affected with these conditions. The questions and their answers were reviewed and discussed over two subsequent meetings among the workgroup and served as a platform to establish clinical practice guidelines in both the affected pediatric and adult populations. A draft manuscript was subsequently circulated among the workgroup for review and editing and was followed by a third meeting for final edits as deemed appropriate. Guidelines in this manuscript focus on the immune system and are based on literature review of over 100 clinically relevant publications, as well as the collective experience and majority consensus of the workgroup members. Content includes not only addressing the T cell compartment, but also B cell abnormalities, the latter of which may predispose affected individuals to infections later in childhood and into adulthood. These guidelines are directed mainly toward individuals with 22q11.2del/DGS. Although data is limited, they may also be applicable to other causes of DTD. Background information provided is intended to provide an understanding and rationale for the recommendations involving the diagnosis and management of abnormal thymic development. Cost and access to resources including laboratory testing and clinical follow-up are recognized as important decision-making determinants as well. Given the variation among centers and regions, adjustments, and accommodations are necessary. Given that each case may be unique, clinical decisions must be based on the individual patient.

## Background

The thymus is an organ responsible for and essential to the production of T lymphocytes. Numerous defects are known to adversely affect thymic development. DGS is historically the condition clearly associated with a small or absent thymus. The small deletion within chromosome 22 was linked to DiGeorge syndrome in the early 1980s [1], although this syndrome was named in 1965 when Angelo DiGeorge described the common embryologic derivation of the heart, thymus, and parathyroid glands [2]. The classic phenotypic triad of DGS consists of conotruncal heart defects, hypocalcemia due to hypoparathyroidism, and T cell deficiency due to thymic hypoplasia [3, 4]. Other sources list five major phenotypic abnormalities, adding abnormal facies and velopharyngeal insufficiency with submucosal cleft palate to the above three criteria [5].

Over the years, the terminology evolved but also proved confusing, due to the fact some individuals have the deletion without evidence of the syndrome, while others meet the clinical criteria for DGS but do not have an identifiable deletion. The term chromosome 22q11.2 deletion syndrome is now used in individuals identified as having a hemizygous deletion of chromosome 22q11.2 [2]. Numerous other gene defects as well as environmental exposures have been identified as causing DTD, also termed thymic hypoplasia. These factors are further detailed in the genetics section.

## Thymic Development

The thymus is responsible for the development of T lymphocytes in utero and after birth. This organ is derived from the third branchial pouch [6]. Development begins around the 4th week of fetal development, with lymphoid stem cells starting to populate the thymus by week 8 of fetal development [7]. Interactions between these developing T cell precursors and the thymic epithelial cells are critical for ongoing thymic development [8]. Incomplete migration of the thymus may result in thymic tissue settling in an aberrant location, anywhere between its point of origin in the high cervical region [9] to its intended destination in the anterior mediastinum. In pediatric necropsy samples of individuals with DGS, thymic tissue was located in various locations along its descent pathway as high as the base of the skull, medial to the submandibular salivary glands, and adjacent to the thyroid gland [10]. Identification of these small rests of ectopic thymus may help explain why a thymus may be present but not visible on imaging or even during cardiothoracic surgery in individuals with 22q11.2del.

The quantitative number of T lymphocytes measured in the blood of individuals with 22q11.2del largely reflects the thymic output and thymic size [11, 12], particularly in the neonatal period. Thus, barring a specific T cell defect such as SCID, a normal thymic volume in 22q11.2del, results in a normal number of T cells, whereas a small thymus can result in a quantitative T cell deficiency. An estimated 67–80% of individuals affected with 22q11.2del have some degree of T cell lymphopenia (TCL) [13]. Studies suggest that approximately 0.5% of those diagnosed with 22q11.2del have a severe immune deficiency with very few T cells due to the absence of a thymus at birth, termed congenital athymia [14, 15]. From this point forward in these guidelines, DTD will be the abbreviation used to encompass the multiple known causes of defects in thymic development that result in thymic hypoplasia.

## Genetics of 22q11.2 Deletion Syndrome

22q11.2 microdeletion syndrome results from a deletion of chromosomal material within the 22q11.21 band, found at the proximal part of the long arm of chromosome 22 [16]. Loss of function of one gene copy, or haploinsufficiency, can lead to an abnormal phenotype. The referral to 22q11.2del as a microdeletion is related to the missing genetic information being too small to be visualized on standard G-banded karyotype analysis. Over 90% of 22q11.2 deletions are de novo (spontaneous), with neither parent affected [17, 18]. In the almost 10% of individuals where a mutation is identified in a parent, the prevalence is nearly equally divided between mothers and fathers [18, 19].

**Fig. 1:**
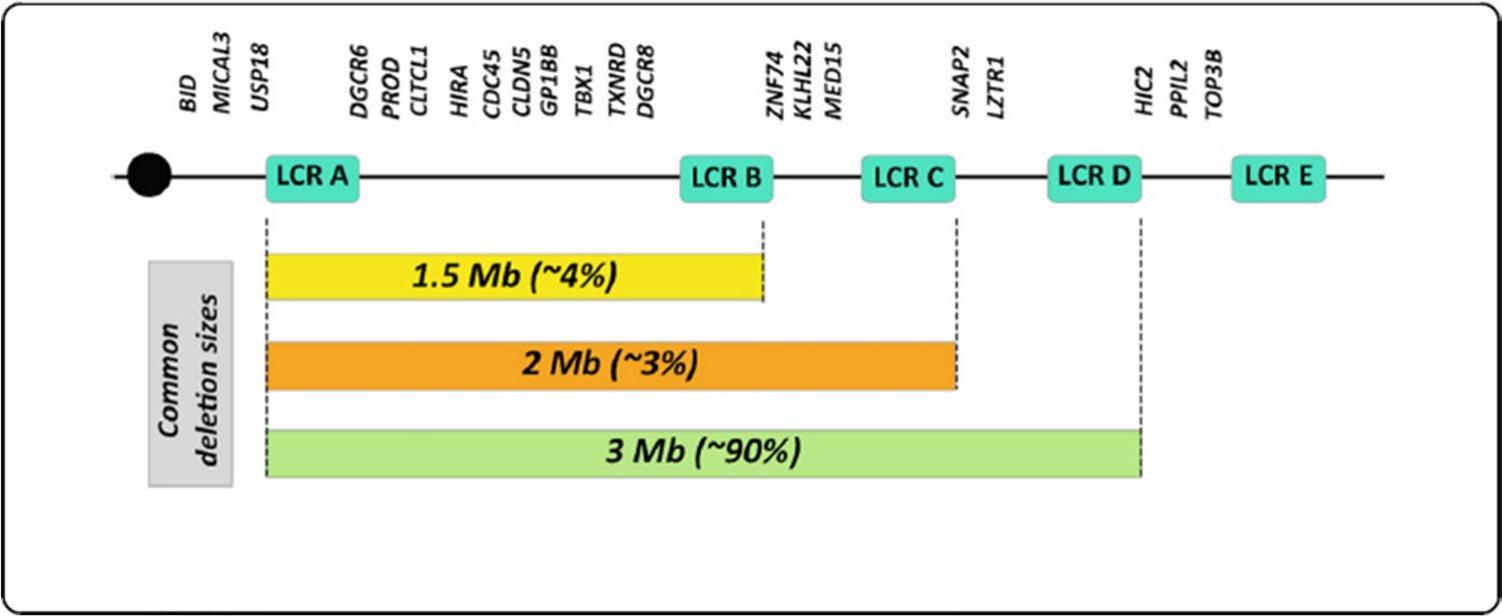
Chromosomal deletions in the 22q11.2 region. Credit Rozas MF, Benavides F, Leon L, Repetto GM. Orphanet J of Rare Dis https://doi.org/10.1186/s13023-019-1170-x

The prevalence of this chromosome 22q11.21 microdeletion is estimated to occur between 1 in 3000 to 1 in 6000 live births, making it the most common chromosomal microdeletion syndrome [20]. The reason for this high frequency is related to the fact that this region contains a cluster of low-copy-repeats (LCRs), referred to as LCR22A-H [21]. These LCRs mediate meiotic non-allelic homologous recombination and are susceptible to either deletion or duplication of these intervals. The 3 most common deletion sizes are 3 Mb (approximately 45 functional genes) [22], 2 Mb, and 1.5 Mb (24 genes) [23]. These deletion sizes correspond to deletions flanked by low copy repeats (LCR) designated A-D, A-C, and A-B, respectively (Fig. 1) [24]. Both LCR A-B and A-D deletions result in similar phenotypes [21], suggesting the major causative genes may be in the LCR-A-B region, though modifier genes beyond LCR-B may partially contribute to the phenotypic diversity [24]. Deletions spanning LCR-C-LCR-E are classified as distal deletions of 22q11.2del [21]. Because distal deletions do not include the *TBX1* gene, the phenotype may not be classic, and individuals are less likely to have associated TCL [25]. Evidence in mouse models suggests that the impact of a *Tbx1* mutation on thymic development is determined by the time point of the deletion. Early homozygous deletion of *Tbx1* in a mouse model results in congenital athymia, whereas mid-stage deletions result in thymic hypoplasia, while a late deletion has no impact on thymic development [26]. This finding may help explain in humans why some patients identified with 22q11.2 microdeletion are phenotypically normal, while others are severely affected with congenital athymia.

## Establishing a Diagnosis/Diagnostic Criteria

The diagnosis of 22q11.2del can be made by various techniques. Fluorescent in situ hybridization (FISH) or chromosomal microarray (CMA) has been the traditional methods of detection. FISH employs a locus-specific probe that is complementary to a particular area of the 22q11.21 region. FISH is specific for identification of 22q11.2 deletion but may not detect individuals with an atypical or distal deletion that does not include the more proximal part of the commonly deleted region (LCR A-B). CMA involves the use of a microarray platform containing DNA probes to detect chromosomal copy number imbalances across the genome [27]. CMA offers several advantages over FISH. It more reliably detects *duplications* of the 22q region versus FISH. CMA also interrogates for deletions and duplications along the rest of the genome, allowing for identification of other chromosomal abnormalities, not limited to 22q11.2del. These features have made it the preferred diagnostic modality for the investigation of a possible chromosome abnormality in most clinical scenarios among clinical geneticists [28, 29]. The two CMA techniques in common clinical use are comparative genomic hybridization-based arrays and single-nucleotide polymorphism (SNP) arrays [30]. While either CMA technique can effectively detect 22q11.2del, technical advantages have led to the SNP array being favored in many major academic centers. Another testing modality used in some centers to detect the deletion is a rapid PCR-based test called multiplex ligation-dependent probe amplification. Genetic panels, whole-exome sequencing (WES) and whole-genome sequencing (WGS), are increasingly used to diagnose primary immune deficiency disorders, including 22q11.2del [31]. However, rather than assessing for the full 22q11.2 chromosomal deletion, which typically involves dozens of genes, targeted gene panels only assess for only a specific single gene deletion such as *TBX1*. Thus, unlike CMA, genetic panels do not determine the extent of a chromosomal deletion. Detection of 22q11.2 microdeletions has also shown promise for use at birth by utilizing multiplex ligation-dependent probe amplification on DNA from neonatal dried blood spot samples [32].

## Prenatal Screening

Prenatally, screening for fetal chromosomal abnormalities using cell-free DNA from a maternal blood sample has become common. Although not recommended in the 2020 American College of Obstetricians and Gynecologists Practice Bulletin [33], many testing companies include an option for microdeletion testing as part of their screening test. The positive predictive value for microdeletion disorders such as 22q11.2del is much lower than for common trisomies, however. Thus, any positive screening test should be confirmed by a diagnostic test, such as CMA or FISH, which could be performed through amniocentesis or postnatal peripheral blood sample.

## Other Genetic Causes of Defects in Thymic Development/Thymic Hypoplasia

When individuals are believed to have a DTD, but test negative for 22q11.2del, other potential genetic causes as well as environmental exposures should be sought to explain the underlying cause. Mutations or deletions in several other genes have been described that impact thymic development and may result in varying degrees of thymic hypoplasia including congenital athymia [34]. These genes include *TBX1*, *CHD7*, *FOXN1*, *FOXI3*, *PAX1*, *TBX2*, and *FOXI3* [5, 35–37].

### TBX1

T box transcription factor 1 (*TBX1*) regulates the expression of transcription and growth factors of the heart, thymus, parathyroid glands, and palate [38] and thus affects the early phases of pharyngeal pouch formation and subsequent thymic development [39, 40]. Mutations in *TBX1* result in a constellation of phenotypic abnormalities. *TBX1* is stipulated to be the primary gene responsible for the phenotypic features associated with 22q11.2del [34, 41], as evidenced by *TBX1* haploinsufficiency correlating with five major phenotypes: abnormal facies, cardiac (conotruncal) defects, thymic hypoplasia, velopharyngeal insufficiency with submucosal cleft palate, and hypoparathyroidism [5]. This effect has been demonstrated in homozygous *Tbx1* knockout mice [39], whereas humans presenting with these features have been identified as having a single mutation in *TBX1 *[5]. Further evidence is suggested by mutations in the *TBX1* binding domain being associated with the DGS phenotype in the absence of the 22q11.2 microdeletion [5]. In one study, 96% (225 of 235) of patients with clinically diagnosed DGS had a defined 1.5 to 3 MB deletion involving the 22q11.2 locus. Of the remaining 10 patients, six had an isolated monoallelic mutation in *TBX1* [5]. Unlike 22q11.2del, intellectual and developmental delay is not associated with an isolated *TBX1* mutation [5].

### TBX2

There are 17 identified human T box genes, each functioning as critical transcriptional repressors and/or activators during embryonic development. In a small case series, three of four individuals identified as having a mutation or deletion of *TBX2* (T box transcription factor 2) had T cell abnormalities [42]. All three were related, two were siblings, and both had been diagnosed with DGS. One had a severe T cell deficiency and met the criteria for athymia at age 7 years. Another had T cell lymphopenia, and a third was described as having low naïve T cells with no TCL. This suggests that *TBX2* may also be associated with thymic hypoplasia.

#### *CHD7* and Charge Syndrome

Chromodomain helicase DNA-binding domain protein (CHD) genes regulate changes in chromatin structure during recombination, transcription, repair, and replication [43, 44]. Coloboma, heart defect, atresia choanae, retardation of growth and development, genital hypoplasia and ear anomalies/deafness (CHARGE) syndrome is most often associated with a mutation in *CHD7*, with 60–70% of patients with CHARGE syndrome having an autosomal dominant mutation in *CHD7* [45–47]. Similar to 22q11.2del, CHARGE syndrome typically occurs sporadically and affects midline development [34, 41]. Clinical features of CHARGE syndrome can overlap with those of 22q11.2del, including cardiac and ear anomalies, hearing loss, cleft palate, developmental delay, and renal anomalies [41]. Facial nerve palsy, tracheoesophageal fistula, and male genital hypoplasia were found to occur with greater frequency in mutation of *CHD7* versus 22q11.2 deletion syndrome [41]. CHARGE syndrome is also associated with TCL secondary to thymic hypoplasia and has been severe enough to require thymic implantation in a small number of patients [41]. A study that included 25 children with CHARGE due to *CHD7* mutation identified 60% as having lymphopenia [41]. Defects in humoral immunity can also occur with this condition [41]. Taken together, this information supports the importance of an immunological evaluation in all patients with CHARGE syndrome. Mutations in *SEMA3E* on chromosome 7q21.11 have also been associated with CHARGE syndrome [48]. This particular gene should be assessed in patients meeting the CHARGE clinical phenotype without a pathogenic *CHD7* mutation [13].

#### *FOXN1* Deficiency

Forkhead box N1 (*FOXN1*) is a transcription factor involved in the development, differentiation, and maintenance of thymic epithelial cells [34, 49–51] and the growth and differentiation of skin epithelial cells, including hair and nails [52]. Normal interactions with the cortical thymic epithelial cells (cTECs) result in positive selection of T cell precursors. These T cell precursors then travel to the thymic medulla and undergo negative selection through interactions with the medullary thymic epithelial cells (mTECs) [53, 54].

*Autosomal recessive FOXN1* deficiency results in an inability of T lymphocyte precursors to interact with cTECs and mTECs [13], leading to profound TCL. Affected individuals also have alopecia and dysplastic nails (particularly affecting the toes). Biallelic *FOXN1* deficiency has been associated with congenital athymia in a small subset of patients who test negative for 22q11.2 deletion. Bone marrow transplantation did not prove effective for children affected with biallelic *FOXN1* deficiency [55]. In 2011, two children with homozygous mutations in *FOXN1* underwent thymic transplantation with subsequent development of naïve T cells [36].

*Heterozygous FOXN1* mutations may be identified after an abnormal TRECs assay when performed as part of the NBS. Affected individuals may have a lesser degree of TCL versus biallelic loss. Congenital athymia is generally not present, and individuals have normal or almost normal appearing hair with only subtle nail dystrophy (spoon nails). Phenotyping often shows CD8 lymphopenia with a normal or mildly decreased CD4 count, with the latter typically improving with time [56]. The TRECs assay on the NBS may be abnormal, and a thymic shadow may or may not be visible on chest X-ray [56]. Patients with a heterozygous *FOXN1* mutation identified as having TCL should be followed clinically and with periodic flow cytometry [13].

### FOXI3

Forkhead box I 3 (*FOXI3*) transcription factor may be another key modulator of thymic development [57]. This recently described candidate gene has been associated with an abnormal TRECs on the NBS and thymic hypoplasia, as well as facial dysmorphism and hypocalcemia in some affected individuals. It has been detected in patients with a microdeletion at chromosomal 2p11.2 [58, 59].

### *PAX1*

Paired box 1 (*PAX1*) is another transcription factor involved in the development of the third pharyngeal pouch and thus also plays a role in T cell maturation and normal thymic development [60]. A non-functioning gene may result in severe T cell lymphopenia with normal numbers of B and natural killer cells. Otofaciocervical syndrome type 2 (OFCS2) can be associated with *PAX1* defects. This autosomal recessive disorder is characterized by facial anomalies, abnormal external ears, preauricular fistula or pits, hearing impairment, branchial cleft, vertebral anomalies, and mild intellectual disability [61].

#### 10p13-14 Deletions

In the 1990s, a few case reports were published describing patients diagnosed with DGS and a deletion at the 10p13-14 locus [62, 63], which was at the time labeled the DGSII locus. Since then, evidence has accumulated that 10p deletions are associated with *GATA3* haploinsufficiency, which can result in hypoparathyroidism, sensory neural hearing loss, and renal dysplasia [64]. Other associated 10p deletion findings include heart defects, delayed language development, and intellectual disability [13]. A DGS expert who reviewed 23 papers on 10p deletions found that T cell or thymic defects were uncommonly associated with this condition [13]. Another study found no 10p microdeletions among 162 patients with suspected DiGeorge syndrome, and thus, it was determined that screening for 10p microdeletion among DiGeorge patients is not indicated [3].

#### Trisomy 21

The incidence of trisomy 21, also referred to as Down syndrome, is high, affecting one in 1200 newborns [65]. The thymus in individuals with trisomy 21 is reduced in size, hypocellular, and contains a decreased proportion of phenotypically mature thymocytes vs healthy controls [66]. These findings are seen even in infants. Thymic biopsies in this population have demonstrated abnormal architecture with accelerated maturation kinetics and premature involution, with early degeneration of Hassall’s bodies [66]. The thymi of affected individuals have been shown to lose their function early in childhood versus the age-related involution that would otherwise occur after puberty [66]. These findings may partially explain why patients with trisomy 21 often have TCL and increased risk for severe and recurrent infections.

## Non-genetic Causes of Thymic Hypoplasia

Biological mothers of individuals with DTD should be asked about potential teratogens, including diabetes, exposure to isoretinoin (retinoic acid) [2], and alcohol consumption during pregnancy.

### Diabetic Embryopathy

Some infants of diabetic mothers (IDM) have congenital athymia in the absence of any identifiable genetic defect [67, 68]. The underlying mechanism is not clearly understood, although some experimental studies suggest that hyperglycemia is teratogenic in diabetic pregnancies.

### Retinoic Acid Embryopathy

Retinoic acid (isotretinoin) represses *TBX1* expression and is a known teratogen associated with a wide spectrum of birth defects involving craniofacial and cardiac malformations [69]. Fetal exposure to retinoic acid has been linked to defects in thymic development including congenital athymia [70, 71].

### Maternal Alcohol Consumption

Clinical features were described with characteristic features of both fetal alcohol and DiGeorge syndrome including facial and immune abnormalities [58, 72]. Studies in murine models have demonstrated that ethanol exposure adversely affects thymic development [73–75].

## 22q11 Duplication Syndrome

The 22q11.2 duplication syndrome was first reported in 1999. It is less well-characterized versus 22q11.2del and estimated to occur at a frequency only half that of the deletion syndrome [76]. As with 22q11.2del, 22q11.2 duplication is also widely variable phenotypically, with many asymptomatic individuals reported [76]. It has, however, also been associated with some manifestations similar to 22q11.2del, including cardiac defects, velopharyngeal insufficiency, intellectual and learning disabilities, short stature, and facial dysmorphism [76–78]. In a small case series of seven patients between 3 and 17 years of age affected with 22q11.2 duplication syndrome, all were found to have normal absolute T, B, and natural killer cell numbers, and all 6 patients evaluated for naïve to memory T cells had normal ratios. However, several were diagnosed with humoral deficiencies. Three of six had low switched memory B cells (CD19 + CD27 + , IgM-). In total, two of the seven were determined to have IgG deficiency, while two others had memory-specific antibody deficiency (SAD) due to rapid waning of pneumococcal titers, and due to this, in conjunction with a concerning infectious history, IGRT was recommended [79]. Evidence from this small case series suggests that patients with 22q11 duplication syndrome are at increased risk for development of antibody deficiencies, and that affected patients experiencing recurrent or severe infections should undergo an immune evaluation [79].

## Terminology Updates

### VCFS (Velocardiofacial Syndrome)

Historically, patients were labeled as having DGS if they had hypocalcemia, thymic hypoplasia, and conotruncal cardiac anomalies [80] and VCFS if they demonstrated dysmorphic facies and conotruncal cardiac anomalies [81]. It was eventually recognized that DGS and VCFS had both phenotypic overlap and a common genetic basis, with 90% of each group having a hemizygous 22q11.2 deletion [24, 82–85]. Thus, the term VCFS has largely fallen out of favor and is now generally referred to as 22q11.2del or DGS in instances where the deletion is not detected.

### 22q Deletion Syndrome vs DiGeorge Syndrome

Given the advances in molecular diagnostics, the practice of referring to a condition by its underlying genetic cause has become commonplace in many medical specialties, including immunology. Available information suggests that 90% of patients diagnosed with DGS have a hemizygous 22q11.2 deletion [82, 86, 87]. Thus, when a deletion of 22q11.2 can be confirmed, many specialists now refer to the condition as 22q11.2del rather than DGS. Other centers may label an affected individual to have DGS secondary to 22q11.2del. Other experts have recommended abandoning the term “DiGeorge syndrome” altogether [88], although this proposal makes it difficult when the phenotypic criteria are met but a genetic defect cannot be identified.

### 22q11.2 Deletion/DGS with Variable T Cell Lymphopenia (Partial, Complete, and Atypical DiGeorge)

The terms partial, complete, and atypical DiGeorge syndrome have been used by immunologists for many years to describe the condition as it relates to the subspecialty of immunology. “Partial DiGeorge” is a term used when one had the clinical phenotype of DGS with T cell lymphopenia due to a variable degree of thymic hypoplasia (but not thymic aplasia). Complete DiGeorge was used to describe DGS with very few or undetectable T cells secondary to congenital athymia, and atypical (complete) DiGeorge referred to patients diagnosed with DGS with congenital athymia who developed autologous immune dysregulation (also referred to as an Omenn-like syndrome or autologous GVHD) [13]. The term DiGeorge syndrome has largely been replaced by 22q11.2del when such a deletion is identified, making the use of the above terms less applicable. Additionally, the terms partial, complete, and atypical apply exclusively to immunology. Given this, the fact that this condition affects multiple organ systems and necessitates co-management through integration of numerous specialists and a primary care provider, coupled with the fact that many clinicians have very limited comprehension of these non-descript terms, continuing to use the above terminology is suboptimal.

The workgroup consensus is that the terms partial, complete, and atypical DiGeorge be substituted with more descriptive nomenclature that directly characterizes the degree of TCL and will be universally understood and accepted (Table 1). Affected individuals have either normal quantitative T cell values, TCL (mild or significant), or severe TCL suggesting congenital athymia. When applicable, it is recommended that the term partial DiGeorge is replaced with 22q11.2del followed by the degree of TCL. When an affected individual does not have an identifiable genetic defect but meets phenotypic criteria for DGS, using DGS followed by characterization of the degree of lymphopenia is most appropriate. For an individual found to have another genetic defect or syndrome causing TCL, such as *CHD7* mutation or CHARGE, the precise condition should also be specified in place of 22q11.2del when appropriate. It should be recognized that the degree of TCL can change over time and an individual with congenital athymia may later develop phenotypic manifestations of autologous immune dysregulation (Omenn-like syndrome).

**Table 1:** Proposed terminology of 22q11.2del in association with T cell lymphopenia

Updated terminology	Previous terminology
22q11.2del^a^ without T cell lymphopenia	DiGeorge without T cell lymphopenia
22q11.2del^a^ with T cell lymphopenia (mild, significant)	Partial DiGeorge
22q11.2del^a^ with congenital athymia	Complete DiGeorge
22q11.2del^a^ with congenital athymia and autologous immune dysregulation (Omenn-like syndrome)	Atypical (complete) DiGeorge

^a^Replace use of 22q11.2del with DiGeorge syndrome when the genetic defect cannot be identified (or another specific causative genetic defect if distinct from 22q11.2del)

## Immunological Laboratory Assessment

### Initial Laboratory Assessment

The presence or absence of an immune deficiency cannot be assessed based on the clinical phenotype of 22q11.2del or CHARGE syndrome [22]. Overall, between 67 and 80% of patients with 22q11.2del and 60% of patients with CHARGE/CHD7 mutation have some degree of T cell lymphopenia [13, 41]. As a result, any person diagnosed with 22q11.2del, CHARGE syndrome, or other condition associated with DTD should undergo an immune evaluation. The initial immunologic evaluation recommended at the time of diagnosis should include T (CD3, CD4, CD8), B (CD19 or CD20), and natural killer (CD16 or CD56) cell (TBNK) quantitation plus assessment of naïve (CD45^+^CD3/4/8^+^RA^+^) and memory (CD45^+^CD3/4/8^+^ RO^+^) T cell subsets (Table 2). When available, results of the T cell receptor excision circles (TREC) assay on the NBS should be reviewed, with an abnormal value increasing the likelihood of significant TCL. At least 3 weeks following the third DTaP administration, a repeat assessment of lymphocyte subset quantitation (TBNK) along with IgG, IgM, IgA, and tetanus IgG levels are recommended. This assessment should generally be undertaken between 8 and 11 months of age, and prior to the live MMR and varicella vaccines at 12 months (see the “Immunization” section for further details). T cell proliferation assays are of limited value as discussed further.

**Table 2:** Recommended periodic laboratory evaluation in 22q11.2del and other DTD in patients with no TCL or mild TCL†

	At diagnosis	8–11 months	Age 4–5 Y^a^	Age 10 Y	Every 5–10 Y
TBNK	X	X	± ^b^		
CD45RA^+^CD3/4/8^+^	X				
IgG		X	X	X	X
IgM		X	X	X	X
IgA		X	X	X	X
Tetanus IgG		X	X	X	±
Pneumococcal serotypes [23]			± ^b^	± ^b^	± ^b^

^a^Obtain at least 3 weeks following DTaP/MMR and varicella boosters but sooner if unable to receive initial live vaccines

^b^Decision to obtain is discretionary, with the need determined by other lab results, infection history, access, cost, cardiac surgery with partial thymectomy performed after 8–11 months, and shared decision-making

^†^Individual management of patients is essential. More frequent or more in-depth assessments may be needed in individuals with significant lab abnormalities, recurring, severe or opportunistic infections, or with underlying concerns for autoimmunity

An immunologic assessment in individuals affected with 22q11.2del and other DTD is necessary to characterize immune status and use the information to help assess infection susceptibility. Mild TCL in 22q11.2del and other DTD may be of no clinical consequence, meaning that even though T cell numbers may be slightly below the reported reference interval, it may not result in affected individuals having increased risk for recurrent, refractory, severe, or unusual infections [89]. This notion contrasts with affected individuals identified as having significant TCL, which may be more likely to increase susceptibility to infections. Results guide recommendations regarding the need for special precautions or interventions. The most immediate need of the initial evaluation is to rule out congenital athymia, as these patients suffer from profound immune deficiency and require immediate isolation precautions and eventually a thymic implant [90]. Failure to diagnose this condition early in life and institute measures to prevent infections can be fatal. Immunological lab assessment also helps determine the safety of live vaccinations, infection susceptibility, the need for prophylactic antibiotics including to prevent pneumonias related to severe T cell deficiencies (*Pneumocystis* or atypical mycobacterial), and how often immunology follow-up may be needed.

### Frequency of Immunological Assessment

Expert opinion has been divided regarding the follow-up needed in the absence of clinical infection [22]. Some clinicians obtain an initial immunologic lab set at the time of diagnosis or initial evaluation and, if normal or deemed unremarkable, recommend immunology follow-up only on an as-needed basis, largely depending on the clinical course. This practice contrasts with the approach of other clinicians who recommend serial immunological evaluations including lab studies in the first year of life then annually thereafter — even when the initial lab sets are normal and one’s infection history is unremarkable. Most approaches fall in between these practices. General guidelines published in 2011 for managing patients with 22q11.2del recommended immunologic evaluations at diagnosis, 0 to 12 months, and 1 to 5 years [22]. The rationale for the updated consensus, shown in Table 2, is largely due to data suggesting that immune function changes over time in 22q11.2del, with the humoral deficiencies being recognized more frequently later in childhood and into adulthood. Recommendations suggest an evaluation at the time of diagnosis and prior to 12 months of age (assuming initial diagnosis is made early in life). Subsequent immune evaluations are recommended following the 4–5-year booster series (including MMRV) and every 5–10-years thereafter, guided by clinical course and patient or family preference. Additional follow-ups are recommended sooner or in between the regularly recommended intervals should recurrent, severe, unusual, or refractory infections develop or for other parental concerns related to immunology or allergy.

### Specific Laboratory Evaluation

T lymphocyte levels vary by age, and reference intervals vary from one lab to another, and no universally accepted definition exists to distinguish mild versus significant TCL. Some experts find it helpful to categorize immune status based on the degree of T cell deficiency, as it aids in the determination of the safety of live vaccines and may help stratify risk for infections or possibly predict a longer duration of infections when significantly decreased. In 22q11.2del/DTD, the degree of TCL can be categorized as none, mild, significant, or severe (congenital athymia/thymic aplasia). The workgroup determined CD4 count of < 400 and CD8 counts < 200 cell/mm^3^ to be significant (Table 3). These values were chosen in part because they are generally the cutoffs used to help ensure safety of live vaccines in infancy (see “Vaccine Recommendations”).

**Table 3:** 22q11.2 deletion/DiGeorge syndrome/DTD—categorization of T cell deficiency

T cell deficiency	TREC assay	T cell deficit	T cell quality
(1) None	Normal	None	Normal
(2) Mild	Normal	Mild	Normal
(3) Significant	Normal or abnormal	Moderate	Normal (usually)
(4) Severe	Abnormal	Severe	Variable/not measurable

*TREC*, T cell receptor excision circle

Normal or adequate T cell immune function in association with 22q11.2del/DTD is suggested by:

1. Absent or mild T cell deficiency
2. Normal/unremarkable proportions of T lymphocyte subsets (CD3^+^, CD4^+^, CD8.^+^, as well as naïve to memory ratio (particularly in the CD4 compartment))
3. Evidence of protective tetanus IgG level at least 3 weeks after the 3rd DTaP (surrogate marker reflecting adequate T cell function)

### Flow Cytometry

The thymus is the only organ where thymocytes can mature, be selected, and develop into naïve T cells [91]. Flow cytometric analysis is required to analyze T cell subsets in the blood and allows for the rapid assessment of severe TCL that might suggest congenital athymia. In this condition, naïve T cell counts should be < 50/mm^3^, and B and NK cell counts are expected to be normal or near normal [13]. A predominance of CD45RA^+^ naïve T cells makes congenital athymia very unlikely. This testing is of paramount importance in the initial diagnostic steps, as severe TCL is considered an immunologic emergency that requires implementation of precautions to protect against infection. Lesser degrees of thymic hypoplasia, if present, and other possible causes of TCL including SCID and less severe forms of Combined immune deficiency (CID) must also be considered. When thymic tissue is present, T cell numbers that are initially low typically increase over the first year of life, and the predominance of CD45RA^+^ naïve T cells continues [13]. When laboratory assessment in the first year of life meets criteria for safe live vaccine administration and ones’ infectious history remains unremarkable, the ongoing need to obtain periodic flow cytometry may not be necessary.

### Recent Thymic Emigrants (RTEs)

**Fig. 2:**
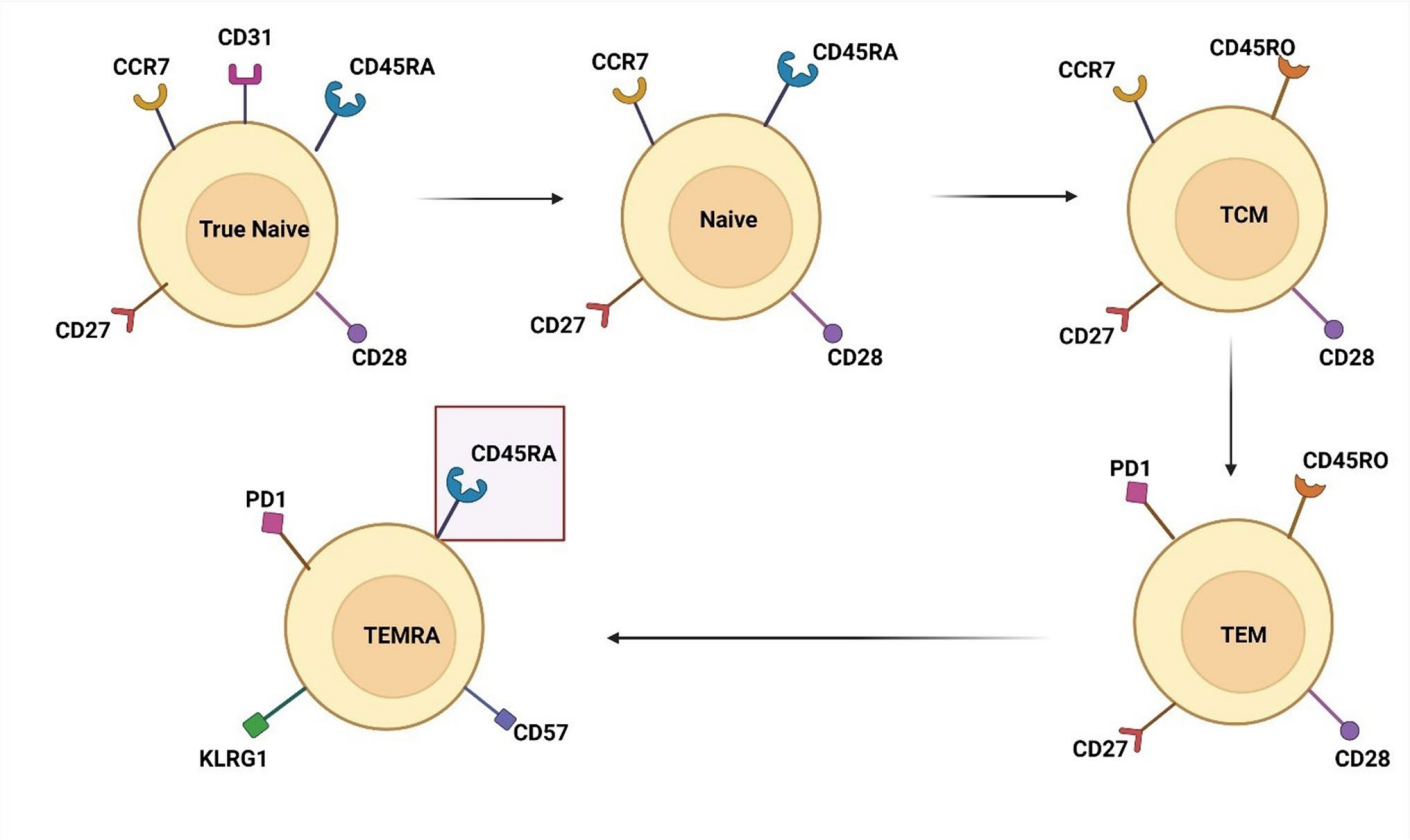
Depiction of how CD45RA may be re-expressed (highlighted in figure) despite not representing a truly naïve T lymphocyte. Unlike TEMRA cells, true naïve recent thymic emigrant T cells express CD31 and CCR7 surface markers. Gated from CD45^+^CD3^+^ T cells expressing either CD4 or CD8. CCR7, chemokine receptor 7; PD1, programmed death 1; LKRG1, killer lectin inhibitory receptor 1; TCM, T central memory; TEMRA, T effector memory re-expressing CD45RA; TEM, T effector memory. Figure created using BioRender.com

Measurement of CD45RA on T lymphocytes has been traditionally used to identify naïve T cells of thymic origin that are by definition antigen inexperienced. However, advances in immunophenotyping indicate that CD45RA is not necessarily an exclusive marker for antigen naïvete of T cells due to the potential for CD45RA to be re-expressed on memory T cells, resulting in the T effector memory re-expressing RA (TEMRA) phenotype (Fig. 2). This re-expression causes the RA marker to be detected by flow cytometric analysis, even though these cells do not represent true naïve T cells. Thus, measurement of CD45RA on T cell subsets may rarely be insufficient if used alone without including a marker of a “true-naïve” T cell. When a TREC assay is not available, markers identifying truly naïve T cells include CD31, CCR7, CD62L, and CD27 on CD4^+^ T cells.

### T Cell Receptor Excision Circles (TREC) Assay

A normal TREC assay functions as an excellent marker for true thymic-derived naïve T cells or RTEs. This assay, which is available on the newborn screen (NBS) in some countries, measures circulating remnants of the DNA excision byproducts formed during V(D)J recombination of T cell receptors in the thymus. It directly reflects the presence and degree of thymic T cell production [92]. An abnormal TREC assay identifies neonates who may have significant TCL. While intended as a screening test to help rule out SCID at birth, it can also detect congenital athymia and less severe forms of thymic hypoplasia [93]. It can also be found in secondary and syndromic disorders of TCL and in prematurity. TREC levels in naïve CD4^+^ CD45RA^+^ cells are decreased at all ages in individuals with 22q11.2del when compared to controls [94]. When available, the TREC assay has helped obviate the need to consider measurement of recent RTEs, such as CD31, CD62L, or CCR7 to confirm the safety of live vaccines.

### Single Versus Double Positive T Cells

Occasionally in evaluating individuals affected with 22q11.2del, an increased number of double positive (CD4^+^CD8^+^) T cells may be detected on flow cytometry. Analysis of thymocyte development has demonstrated numerous perturbed maturation kinetics, sometimes resulting in an accumulation at the double positive (DP) stage [66]. This occurrence may be due to alterations involving the thymic architecture in 22q11.2del, resulting in a reduced ability to attract thymocytes from the cortical to the medullary areas, resulting in a reduced number of thymocytes able to attain single positive (SP) status [66].

### Assessment of T Cell Function/Mitogen and Antigen Stimulation Assays

Even in the presence of TCL, mitogen, antigen, and anti-CD3 T cell stimulation assays are not routinely recommended in individuals diagnosed with 22q11.2del/DTD. The exception is perhaps with congenital athymia when a thymic implant may be necessary, as peripheral autologous oligoclonal T cell expansion manifesting phenotypically as an Omenn-like syndrome can result in high numbers of circulating T cells and substantial mitogen responses despite the fact the T cells are predominantly of the memory phenotype [95]. In this situation, the determination of T cell function may guide the need for immune suppression strategies before and after thymic implantation. In 22q11.2del/DGS, the T cell deficiency is quantitative, secondary to thymic hypoplasia, rather than qualitative (functional), so the presence of T lymphocytes that express predominantly naïve T cell markers early in life, adequate RTEs, or a normal TREC on the NBS provide reassurance that T cell function is expected to be preserved [96–98]. A general consensus was reached among the workgroup that evidence of a protective tetanus IgG antibody level measured at least 3 weeks following the third tetanus (DTaP) immunization (typically recommended at 6 months of age) can serve as a surrogate for T cell function in lieu of obtaining any T cell functional assay prior to receiving MMR and varicella vaccines. This guidance is because tetanus vaccine responses are T cell *dependent* and thus require functioning T lymphocytes to elicit a protein-specific antibody response. Obtaining a tetanus IgG antibody level is a relatively simple, convenient, and cost-effective method to help confirm the presence of adequate T lymphocyte function.

Numerous factors can also make measurement of in vitro T cell assays complicated and even misleading. PHA, ConA, and PWM are plant glycoproteins (lectins) that stimulate T cells to divide through nonspecific binding to the T cell receptor, which can result in a normal PHA response despite a severe T cell functional defect. Likewise, abnormal mitogen responses can exist in the setting of normal in vivo T cell function. This dichotomy is related to the method of assessing T cell proliferative responses and its sensitivity, especially in the setting of TCL. Cellular dilution and a low number of T cells can confound interpretation of mitogen responses especially in severely lymphopenic patients, with results correlating with the sensitivity of the assay used to detect T cell proliferation. Standard mitogen proliferation and even anti-CD3 assays are usually diminished when the T cell count is extremely low, corresponding to the diminished numbers of lymphocytes, specifically T cells, rather than a diminution of function [85, 94]. These limitations can potentially be overcome using flow cytometry-based assays. A general problem is that in vitro T cell functional assays are costly and require maintenance of viability, which can make the time of drawing, processing, and shipping arrangements challenging. These assays are also performed in a very limited number of laboratories and thus frequently require overnight shipping. Especially when transported across long distances, specimens are more likely to be affected by temperature extremes and delays, which can affect cell viability and thus T cell function.

### T Cell Receptor V-Beta (TCR Vβ) Repertoire

T cell subsets of patients with 22q11.2del and TCL have shown restricted TCR diversity [93]. Affected individuals have been shown to have both oligoclonal populations as well as loss of certain TCR V-beta (Vβ) families in the T cell repertoire [99]. In one study, complementarity determining tegion-3 (CDR3) spectratyping has shown restrictions of TCR Vβ repertoires in 54% of CD4 subsets and 60% of CD8 subsets which also correlated with reduced (but not absent) TREC levels [93]. Despite these findings, the patients in this study had a normal PHA T cell proliferative response and no history of recurrent or opportunistic infections. In cases of congenital athymia, the TCR Vβ repertoire would be expected to be severely restricted if detectable at all. This test may be most effectively utilized when concern arises for congenital athymia in an individual with autologous T cell expansion (oligoclonality), even with a response to mitogens [95]. This phenomenon may be seen in cases of 22q11.2del with congenital athymia and autologous immune dysregulation (Omenn-like syndrome) when the patient would be expected to have severe T lymphocyte oligoclonality due to peripheral expansion of pre-existing T cells [95]. When comparing TCR Vβ assessment by flow cytometry versus spectratyping, the former is useful for broad screening and is more widely available, although most commercially available antibodies cover approximately 65% of the TCR Vβ repertoire. Spectratyping covers all [23] known families of the TCR Vβ genes, making it a more comprehensive analysis.

### Testing for SCID and Other Causes of Severe T Cell Lymphopenia

When patients present with severe TCL and very low naïve T cells, it is critical to differentiate congenital athymia from SCID, as the potential treatments are very different. Although exceedingly rare, there are at least two case reports of individuals identified as having 22q11.2 deletion with congenital athymia in addition to SCID, which would render a thymic implant alone inadequate for survival [100]. Each of these patients had 22q11.2del in addition to a SCID-causing Artemis mutation [100]. Confirmation of normal B and NK cell representation on flow cytometric analysis could significantly reduce the likelihood of SCID associated with a B and/or NK cell deficiency. Genetic defects associated with the T^−^B^−^ SCID phenotype include *ADA*, *RAG1/2*, and DNA double-strand break repair genes (e.g., Artemis, DNA-dependent protein kinase catalytic subunit (DNA-PKcs), DNA ligase IV). Equally important is recognizing that genetic defects involving IL-7R alpha and CD3 chains cause T^−^B^+^NK^+^ forms of SCID, potentially making it not possible to rule out as co-existing with 22q11.2del and congenital athymia unless additional data is obtained. For these reasons, when an individual is believed to have thymic aplasia, and thymic implantation is being considered, genetic testing for other causes of severe TCL including SCID is recommended, even if a deletion of 22q11.2 is confirmed.

## Age-Related Immune Abnormalities

In neonates diagnosed with 22q11.2del, the immune assessment is focused on the T cell compartment. Similar to unaffected individuals, in 22q11.2del, one would expect the T cell counts to increase over the first year of life and then begin to gradually decline [96]. Specific to 22q11.2del, T cell numbers may increase more rapidly over the first 6 to 12 months of life and have been shown to have a slower rate of age-related decline compared to unaffected individuals. A study of almost 200 patients found that between infancy and 9 years of age, the average decline in CD3 counts was 25 cells/mm^3^/year (versus 144 in controls), CD4 counts declined 23 cells/mm^3^/year (versus 118 in controls), and CD8 counts 3 cells/mm^3^/year (24 in controls) [96]. Only the difference in CD8 counts was not statistically significant. This slower decline in individuals with 22q11.2del helps explain why T lymphocyte numbers tend to approach normal levels by late childhood/early adulthood [2, 101]. It is uncertain how much of this compensatory mechanism may be explained by modified thymic involution, an increase in peripheral expansion (homeostatic proliferation), or other causes [94, 96].

Patients with 22q11.2del can experience an accelerated decline of CD45RA and an accelerated increase in CD45RO in both the CD4^+^ and CD8^+^ T cell compartments [66, 94]. These changes may be due to impaired thymic production of naïve cells, increased peripheral expansion of CD45RO^+^ cells, or accelerated conversion of RA to RO phenotype due to infections [94]. The progressive exhaustion of naïve T cells and skewing toward a memory phenotype may partly explain recurrent infections in these patients [66].

## Infections in Later Childhood and Adulthood

For many years, the focus of the immune deficiency in 22q11.2del revolved around the TCL in infants and very young children. Survival into adulthood has become the norm [102], which is largely credited to advances in the ability to correct the congenital heart disease that often accompanies this condition. With now many more affected adults, conditions more likely to develop later in life are increasingly being recognized. These problems include both ongoing and new immunological issues. Evidence now demonstrates that abnormalities related to the B cell compartment in these patients may increasingly explain recurrent infections with increasing age. Recurrent sinusitis, otitis media, and lower airway infections continue to occur in 25 to 30% of individuals over 9 years of age and into adulthood [103].

## Humoral Immune Deficiency in 22q11.2del

In individuals with 22q11.2del with only mild to moderate T cell lymphopenia, T cell numbers do not predict susceptibility to infections [69, 87]. Immunoglobulin and other humoral abnormalities in 22q11.2del have been increasingly recognized in recent years [97, 104]. Even individuals whose immune evaluation may be normal early in life are at increased risk for development of IgG deficiency, which may progress to a clinical and biologic picture analogous to CVID [97]. Individuals who experience recurrent infections are much more likely to demonstrate humoral abnormalities [105]. In 855 patients with DGS through the US Immunodeficiency Network (USIDNET) and the European Society for Immunodeficiencies (ESID), 42% who had a confirmed 22q11.2del, IgG, IgA, and IgM abnormalities were examined [97]. Investigators identified 6.2% of patients over age 3 years and 5.6% over age 5 years with an IgG level < 500 mg/dL. Two percent of the cohort had IgA levels < 5 mg/dL, and 23% had IgM levels < 40 mg/dL, all at greater than 3 years of age [97]. Overall, 3% of patients in this cohort were receiving immunoglobulin replacement therapy. No clear association was found between low CD3 counts and abnormal IgG, IgA, or IgM levels. Although total CD19^+^ or CD20^+^ B cell counts were normal in the majority of affected patients, non-switched memory B cells (CD27^+^IgM^+^IgD^+^) were significantly decreased [105], whereas isotype-switched memory B cells (CD27^+^IgM^−^IgD^−^) were only slightly reduced [105]. Some evidence suggests that the restricted T cell repertoire could adversely affect T cell activation and B cell differentiation, which may provide an explanation for the humoral abnormalities that may develop over time [99].

These findings have led to the recommendation for patients with 22q11.2del to have periodic evaluations with a humoral immune assessment beginning at 8–10 months of age, regardless of whether earlier immune assessments are normal. Quantitative immunoglobulin assessments should be obtained earlier if the child has early onset of recurrent, unusual, refractory, or severe infections, keeping in mind that the IgG in an infant is predominantly maternally derived early in life, and an undetectable IgA level (< 6 mg/dL) is not considered abnormal in the first year of life. The workgroup recommends obtaining periodic humoral assessments along with a detailed clinical infection history initially every 5 years if clinically well regarding infections and then every 5–10 years. If the infection history is abnormal or if ongoing humoral assessments are abnormal, an additional and more comprehensive immune work-up should be performed and include assessment of capsular polysaccharide-specific responses.

### Specific Antibody Responses

Similar to other primary immune deficiency disorders, in 22q11.2del, normal immunoglobulin levels do not exclude impaired specific antibody responses. In a study evaluating the frequency of impaired specific antibody response to pneumococcal polysaccharide antigens ages 4 to 22 years, 11 of 20 patients (55%) had an abnormal or poor response [104]. All but one of these patients had IgG levels over 550 mg/dL, and 80% were experiencing recurrent infections. The frequency and severity of infections improved following prophylaxis with co-trimoxazole or IgG replacement therapy (IGRT) [104], suggesting these abnormalities are more likely to be clinically significant. If capsular polysaccharide-specific responses are truly T cell independent, this would suggest an underlying defect in the B cell compartment, unrelated to any T cell abnormalities.

## Infection Susceptibility

An estimated 35 to 40% of individuals with 22q11.2del experience recurrent infections [22]. This increased incidence is likely multifactorial, related to anatomical variations, cellular or humoral immune deficiency, or both [96]. In early childhood, prolonged viral respiratory infections with or without secondary bacterial infections are the most commonly described condition [106]. The frequency of these infections does not correlate with T cell counts, which suggests that anatomical causes may be the major contributor to these symptoms [106]. The frequency of infections was assessed in a cohort of 55 individuals with 22q11.2del ages 9 years and older [96]. This age restriction was used to minimize numerous confounders at a younger age, such as daycare, reflux, cardiac surgery, and velopharyngeal insufficiency, complicating upper airway issues and bottle feeding. Recurrent episodes of otitis media, sinusitis, bronchitis, and pneumonia were each defined by more than three episodes per year requiring antibiotics, all within the previous 2 years. Results demonstrated that 40% of the individuals were considered as healthy as their peers. The incidence of recurrent sinusitis was 27%, recurrent otitis media 25%, recurrent bronchitis 7%, and recurrent pneumonia 4%. Two individuals in this group had recurrent parotitis, two had extensive warts, one had mastoiditis, and one had osteomyelitis. Prolonged viral upper respiratory tract infections were common [103]. Interestingly, the frequency of infection did not correlate with T cell counts [69]. Related to COVID, a survey including 25 patients with 22q11.2del and confirmed infection self-reported low rates of severe disease and no deaths [107]. Some individuals with 22q11.2del also experience esophageal motility disorders, which can impair swallowing and predispose to aspiration, leading to recurrent pneumonias. In patients experiencing pneumonias, an effort should be made to determine the underlying cause, with consideration for a fluoroscopic video swallow study under imaging. The authors did not find any significant difference in immunology laboratory parameters when comparing patients experiencing recurrent infections to those who were clinically well [96].

## Anatomical Considerations Predisposing to Infections in 22q11.2del

The abnormal embryologic development associated with 22q11.2del often involves structural facial variations. Features can include smaller sinuses and sinus ostia, narrowed nasal passages, Eustachian tube abnormalities resulting in poor drainage and weakness or structural changes of the upper palate including a submucosal cleft (palpable bony defect in the hard palate), and velo-pharyngeal insufficiency (VPI). Any of these anatomical changes can increase susceptibility to the upper respiratory tract infections involving the ears and sinuses [103]. Palatal dysfunction, present in > 50% of individuals with 22q11.2del, is one of the major contributors to recurrent infections in children [103]. Palatal weakness impairs the ability to close off the nasopharynx and increases susceptibility to nasal regurgitation and recurrent otitis media [103, 108]. Accompanying swallowing dysfunction can result in formula accumulation in the posterior oropharyngeal area, including the vallecula, posing a risk for regurgitation into the Eustachian tubes or sinuses to further increase susceptibility to upper respiratory tract bacterial superinfections [96, 103]. These abnormalities accentuate the need for individuals with 22q11.2del to be managed by an otolaryngologist familiar with this syndrome. This is especially true when a tonsillectomy is being considered, as affected patients are at increased risk of having a medially displaced carotid artery. Performing a tonsillectomy in these individuals is more likely to result in a serious complication [109]. Additionally, in those with VPI, performing an adenoidectomy may be more likely to worsen the condition, as the adenoids can help reduce the degree of insufficiency and nasopharyngeal reflux [109].

## Vaccine Recommendations for 22q11.2del and Other Defects in Thymic Development

It is well established that vaccinations have played an essential role in greatly reducing morbidity and mortality from disease, particularly in children. This fact applies to both immunocompromised and immunocompetent individuals. The response to vaccination in some immunocompromised patients, even though reduced, still reduces risk of complications, hospitalization treatment cost, and even death from wild-type infections [110]. A basic premise in immunocompromised individuals is that it is necessary to consider the potential consequences of administering versus withholding vaccinations [111], as infections due to wild-type viruses may be particularly severe in individuals with TCL [112].

### Inactivated (“Killed”) Immunizations

Inactivated vaccines pose little to no increased risk of harm in immunocompromised individuals. Routine inactivated vaccines in 22q11.2del/DTD are therefore recommended per standard immunization practices. This recommendation generally applies unless they are receiving IGRT, or when it is known they will be ineffective, such as with congenital athymia.

### Vaccination to Prevent *Streptococcus pneumoniae* Infections.

The Center for Disease Control and Prevention has published recommendations regarding the use of the pneumococcal polysaccharide vaccine for children with congenital T lymphocyte deficiency. For an affected child between 2 and 18 years, two doses of the 23-valent pneumococcal polysaccharide vaccine (PPSV23) are recommended. The first one should be given at least 8 weeks following any PCV-13 vaccination, and the second at least 5 years after the first PPSV23. For age 18 and over, if the PCV-20 has been administered, PPSV23 is not indicated *(CDC.gov 1.24.22).*

### Active (“Live”) Immunizations

To date, no prospective study has been conducted, nor are there published evidence-based guidelines on immune parameters for administration of live vaccines (which include rotavirus [oral], MMR varicella) in individuals with 22q11.2del/DTD [13], although retrospective studies have provided insights (below). As a result, practices have varied widely on parameters used to recommend live vaccines. This discrepancy was discussed in considerable detail among the workgroup, with a consensus summary recommending that patients meet four criteria (Table 4).

**Table 4:** Guideline recommendations^a^ for live vaccine administration (MMR and varicella) in 22q11.2del at age 12 months

Laboratory results (blood)	Comment
1. CD4 $\ge$ 400 cells/mm^3^ (absolute)	Recommended
2. CD8 $\ge$ 200 cells/mm^3^ (absolute)	Recommended
3. Tetanus IgG protective (3 + weeks after dose 3) †	Recommended
4. CD45RA^+^CD3^+^/4^+^ % > CD45RO^+^CD3^+^/4^+^ %	Utilize data from earliest assessment
If available and T cell numbers abnormal, consider either confirmation of normal TREC assay result on NBS or flow cytometry confirming RTEs (CD31)	When available, either marker of RTEs can help confirm adequate thymic function AND helps rule out most causes of SCID

^a^Some experts may recommend immunizing individuals in certain situations who may not meet each of the above criteria. *RTE*, recent thymic emigrants

^†^Assessment of hepatitis B IgG surface antibody may serve as a reasonable alternative if tetanus IgG assay is not available

When individuals meet the criteria listed in Table 4, live attenuated immunizations are recommended and can greatly reduce the risk of contracting wild-type disease. This includes the MMR (measles, mumps, rubella) and varicella vaccines. Because the oral rotavirus vaccine series must be administered prior to the ability to assess response to the DTaP series, only criteria 1, 2, and 4 should be met. When appropriate, other live vaccines that can be considered include the BCG, yellow fever, *Salmonella typhi*, nasal influenza, and smallpox vaccines.

### MMR and Varicella Vaccines

When the criteria listed in Table 4 are met, it is recommended that 22q11.2del patients receive the live MMR and varicella vaccines at 1 year of age. These criteria include a minimum CD4 count of 400 cells/mm^3^, with minimum CD8 count 200 cells/mm^3^, naïve/memory immunophenotyping suggesting a predominance of naïve T lymphocytes at some previous time point, and protection against tetanus as evidenced by a protective level obtained at least 3 weeks after the 3rd dose of the DTaP vaccine. The TREC assay, available on the NBS in some countries, measures remnants of T cell receptors made in the thymus during V(D)J recombination and serves as a marker for RTEs, as does flow cytometry for CD31, CD62L, or CCR7. While direct assessment of RTEs is usually not necessary, it helps confirm T cell production in the thymus. If the extent of immune deficiency is severe enough that a thymic implant (transplant) is being considered, immunizations are not recommended.

Several publications have detailed administration of live vaccines in the subset of patients affected with mild to moderate T cell lymphopenia in the setting of 22q11.2del [110, 111, 113–115]. One cohort included a retrospective analysis of 59 patients diagnosed with 22q11.2del, with and without TCL, where 52 received the MMR vaccine and 32 were administered the varicella vaccine. In this study, none receiving either vaccine had been diagnosed with a *severe* T cell deficiency. No patient receiving either live vaccine experienced severe adverse reactions. Importantly, 63% of those not vaccinated against varicella developed wild-type varicella (study included patients seen between 1994 and 2002), while none of the vaccinated children developed wild-type disease. They concluded that in the absence of severe immunocompromise, vaccinating children with 22q11.2del with live viral vaccines does not carry a significantly higher risk of adverse reactions versus the general population and can greatly decrease the risk of contracting wild-type disease [111]. Other safety studies have drawn similar conclusions [112–114].

Support for recommending minimum CD4 and CD8 values for live vaccine administration was highlighted in a 2007 publication involving a case report of a 13-month male with a hemizygous 22q11.2 deletion, who at 8 months of age was found to have significant T cell lymphopenia, with CD3^+^ 396 cells/µl (21%) (normal 2400–6900 (50–77%)): CD4^+^ 320 cells/µl (17%) (normal 1400–5100 (33–58%)), and CD8^+^ 57 cells/µl (normal 600–2200 (13–26%)) [116]. Lymphocyte proliferation to PHA was reported as normal at age 12 months. He inadvertently received the MMR and live attenuated varicella vaccine at 12 months of age. He subsequently required hospital admission for pneumonia, with a BAL specimen showing clusters of giant cell nuclear inclusion bodies and tracheal aspirate PCR positive for the varicella vaccine strain. Vesicular lesions on his trunk were also positive for the varicella vaccine strain. Testing for measles was negative. The patient received 14 days of IV acyclovir but required a prolonged intubation for chronic lung disease and died from a pulmonary hemorrhage at 19 months of age [116]. It is likely his inability to suppress the attenuated varicella strain was related to his profound CD8 lymphopenia rather than the CD4 lymphopenia, though this could not be confirmed as both were significantly decreased.

The FDA package inserts for both the MMR and varicella vaccines list cellular or humoral immune deficiencies as a contraindication to administration despite the fact they may benefit patients with mild or even moderate forms of immunodeficiency [111]. According to the American Academy of Pediatrics Red Book guidelines, all live bacterial and live viral vaccines including rotavirus are contraindicated in the presence of thymic aplasia (“complete DiGeorge”) [117]. When a patient is affected with “partial DiGeorge,” all live bacterial and live viral vaccines are also listed as contraindicated. However, comments in the guidelines suggest that children with CD3 counts ≥ 500 cells/mm^3^ and CD8 counts ≥ 200 cells/mm^3^ and normal mitogen responses could be considered for MMR and varicella (but not MMRV) vaccination. Our workgroup guidelines, which include a CD4 count of ≥ 400, a CD8 ≥ 200, an adequate CD45RA and do not include a recommendation for assessment of mitogen responses, provide the opportunity for more individuals with 22q11.2del to receive live vaccines.

Some experts may recommend live vaccinations for their patients when the CD4 count is as low as 300 and other criteria are met, although the safety of this approach has not been established on a large scale. A prospective study could serve to determine precisely what T cell criteria would render these vaccines safe.

### BCG Vaccine

In countries where tuberculosis has a relatively high prevalence, the live attenuated BCG vaccine is administered soon after birth and thus is the first live vaccine administered. In cases of severe T cell lymphopenia, such as with congenital athymia, administration may result in disseminated mycobacterial disease, which can cause serious morbidity or mortality. Thus, if 22q11.2del/DTD is suspected as a possibility for any reason pre- or postnatally, vaccination with BCG should be withheld until significant T cell lymphopenia can be excluded. Such scenarios include when either biological parent has known or suspected history of 22q11.2del or other defect in thymic development, when suggestive features including abnormal facies or palatal defects are noted, when a conotruncal cardiac anomaly is detected, when no thymic shadow is detected on neonatal imaging (if performed), or when hypocalcemia is identified with no other identifiable cause. If the BCG vaccine is administered, and the patient is subsequently diagnosed with a 22q11.2del/DTD with significant T cell lymphopenia, consultation with an expert in infectious diseases to discuss prophylaxis or treatment with agents such and isoniazid and rifampin is recommended.

### Rotavirus Vaccine

In countries where BCG vaccine is not administered in the first few days after birth, the oral rotavirus vaccine is typically the first live vaccine administered as early as 6 weeks of age. If administered in the setting of significant T cell lymphopenia, including congenital athymia, it may result in prolonged shedding of the attenuated virus in stool and can result in persistent diarrhea. This complication may increase the risk for dehydration and/or electrolyte disorders, malabsorption, or failure to thrive. Depending on the severity of symptoms, intravenous hydration or nutrition may be required. Rarely, attenuated rotavirus vaccine can be life-threatening in susceptible patients.

### Oral Polio Vaccine

The live oral polio vaccine has been replaced by the inactivated intramuscular polio vaccine in many countries. This is at least in part to numerous reports of individuals who developed complications after receiving the live vaccine and were only later determined to have an immune deficiency. Specific to the live oral polio vaccine strain, reversion to wild-type virus years after immunization in this subset of patients is described [110, 118]. An unintended consequence can include paralytic polio and death. No data on the safety of oral polio in 22q11.2del/DTD are available.

### Yellow Fever Vaccine

The yellow fever vaccine is another live attenuated vaccine recommended for high-risk immunocompetent patients, including those who reside in or travel to yellow fever endemic areas. It can be administered as early as 6 months of age and is contraindicated in immunocompromised patients but should be considered when the criteria for administration of MMR and varicella vaccines are met and exposure risk to wild-type disease is significant.

### Nasal Influenza Vaccines

The quadrivalent influenza A and B nasal formulation is also a live-attenuated vaccine, approved for administration as early as 2 years of age. Although no serious adverse effects have been reported when administered in patients with 22q11.2del, data are limited. When the criteria listed in Table 4 are not met, it may be best to instead recommend the inactivated injection.

## Vaccine Recommendations for Close Contacts

To protect 22q11.2del/DTD patients diagnosed with impaired immunity significant enough that live vaccinations may be unsafe or might not induce protective immunity, close contacts should be immunized whenever possible. This practice is particularly important for members who share living space. Rare exceptions when close contacts should not receive live vaccines are related to those associated with viral shedding. This mainly applies to the live oral polio virus, as live vaccines multiply in the host and interact with host cells following administration [110], which has led to horizontal transmission in some patients with compromised immunity. While the live rotavirus vaccine should be held in infants with significant TCL, household contact spreading of the attenuated vaccine strain to infants with SCID has not been reported [114], which makes it likely the risk from horizontal transmission in individuals with 22q11.2del would also be extremely low. Live attenuated nasal influenza vaccine can be given to close contacts because of its low rate of transmission to others [117]*.* If a varicella rash develops in a close contact following any varicella or live zoster vaccine, the risk of transmission to a patient with significant impairment of immunity is minimal unless blisters develop at the administration site [115]. In such an event, separation of the vaccinated individual from the patient is recommended. When not possible, the vesicles should be completely covered, preferably with two layers of bandage or clothing, to minimize risk of contact. If a direct exposure to the lesions is suspected, prophylactic varicella zoster immune globulin should be considered [115]. Antiviral agents such as intravenous acyclovir or oral valacyclovir can be administered in the unlikely event that the patient contracts the attenuated virus through direct contact [115]. No contraindications exist against vaccination with MMR in household members [110].

## Other Immunological Management of 22q11.2del

The majority of patients affected with 22q11.2del are not predisposed to opportunistic infections [94], and most will not require special precautions related to infections, with exceptions described below.

## IGG Replacement Therapy (IGRT)

Most patients affected with 22q11.2del will not require immunoglobulin replacement therapy [13]. In a study that included 855 patients diagnosed with DGS, only 3% were receiving IGRT [97]. The decision to initiate IGRT, in either subcutaneous (SC) or intravenous (IV) form, is typically based on the clinical status related to infection history or susceptibility, supportive laboratory data, and when appropriate shared decision-making based upon parental and/or patient preference. Absolute indications for IGRT include congenital athymia and common variable immune deficiency (CVID) in association with 22q11.2del. Various other B cell abnormalities which may be indications for IGRT include significant IgG deficiency associated with poor specific antibody responses or markedly decreased IgM levels in association with recurrent infections [4]. Specific antibody deficiency (SAD) when severe or in association with serious or frequent infections may also serve as an indication. It is important to recognize that in 22q11.2del, initial low immunoglobulin levels can normalize with time [96], suggesting that patients placed on IGRT at an early age should have periodic immune testing to assess whether there remains a need for continuation of therapy.

## Antibiotics: Early Use and Prophylaxis

When IGRT is not indicated based on laboratory evaluation, not preferred, or not an option based on access or cost, prophylactic antibiotics should be considered in those requiring frequent courses of antibiotics due to recurrent bacterial respiratory tract infections. Prophylactic use of trimethoprim/sulfamethoxazole has shown symptomatic improvement in individuals with 22q11.2del and an impaired response to the pneumococcal polysaccharide vaccine [104]. Alternatively, early initiation of antibiotics at the onset of symptoms of a respiratory tract infection may be considered. This option might be appropriate in cases when viral upper respiratory tract infections (“colds”) are intermittent but frequently progress to either bacterial sinus or ear infections requiring antibiotics to clear, and the patient has evidence of significant immune deficiency or an anatomical abnormality that predisposes to these infections. Preventing the progression to bacterial sinusitis can reduce the severity and duration of symptoms, time out of work, and missed school days. Early initiation of antibiotics may also reduce the overall use of antimicrobials versus daily administration.

## Antimicrobial Prophylaxis

### Pneumocystis jirovecii Pneumonia (PJP)

No formal guidelines exist concerning parameters for initiating PJP prophylaxis in 22q11.2del. Some clinicians use HIV guidelines related to CD4 T cell counts, although the fact that T cell proliferation is typically normal in 22q11.2del likely explains why these individuals do better clinically versus patients with HIV and comparable T cell counts [103]. Unlike with HIV, in 22q11.2del, opportunistic infections are rare unless congenital athymia is present [14]. Thus, significantly lower CD4 counts in 22q11.2del may be acceptable before starting PJP prophylaxis [119]. Trimethoprim/sulfamethoxazole (TMP/SMX 5 mg/kg/day trimethoprim 3 days per week) remains the drug of choice for PJP prophylaxis [120]. Alternative prophylactic regimens include pentamidine 5 mg/kg every 4 weeks, dapsone 1 mg/kg/day, or atovaquone 30 mg/kg/day [120]. PJP prophylaxis is recommended in athymic patients [120].

### Mycobacterium avium Complex (MAC).

*Mycobacterium avium* is widely present in the environment, including in food and water sources. In individuals with HIV, increased risk for MAC disease typically occurs with CD4 < 50 cells/mm^3^. MAC prophylaxis should be considered in 22q11.2del/DTD with congenital athymia. Azithromycin is the preferred prophylactic agent for infants.

## Blood Products and Surgical Interventions

Cardiac anomalies affect 75–80% of individuals with 22q11.2del, and a significant number of these patients will require cardiac surgery at an early age. Whenever possible, the diagnosis of congenital athymia should be determined prior to surgery, as this small subset of patients requires blood products that are irradiated, leukocyte reduced, and CMV negative. The implementation of these measures can help reduce the risk for GVHD as well as transmission of CMV and other blood-borne pathogens. Given its association with 22q11.2del, many institutions obtain an early genetic evaluation in the neonatal period for conotruncal heart defects, especially tetralogy of fallot, truncus arteriosus, and interrupted aortic arch. T cell subsets should be obtained prior to surgery especially if non-irradiated blood products may be administered [121]. Although there are no definitive guidelines on the use of irradiated blood products, recommendations are that neonates and infants with T cell counts below 400 cells/µl, of which less than 30% are naive, should receive irradiated red cells or platelets when possible [122]. Irradiated cellular blood components are also recommended in cases where a T cell immunodeficiency is suspected but an immune evaluation cannot be undertaken prior to surgery.

## Congenital Athymia and Thymic Implant

22q11.2del is the most commonly described genetic defect associated with congenital athymia [34]. Congenital athymia represents a very small subset of 22q11.2del/DGS patients as it occurs in less than 1% of those diagnosed with 22q11.2del. The diagnosis requires a severe deficiency of naïve T cells and is traditionally defined as either CD45RA^+^CD3^+^ T cells co-expressing CD62L < 50 cells/mm^3^ [95] or CD45RA^+^ CD4^+^ T cells < 50 cells/mm^3^ on two separate occasions [121] in the absence of other explainable causes, such as SCID. Characteristic immunologic features of congenital athymia resemble SCID and include profound T cell deficiency with risk of recurrent, severe, or opportunistic infections. In data collected from 105 thymic implants performed at one center over time, 38% had a deletion of 22q11.2, 11% had *CHD7* mutations, 3% had a *FOXN1* homozygous mutation, and 1 each had *TBX1* and *TBX2* mutations. Thirty-nine percent had no genetic mutation identified, although 29 of these 41 patients with thymic aplasia who underwent transplant were infants of diabetic mothers. Survival following implant was 72% (76 of the 105 patients). Twenty three of the 29 deaths occurred within 1 year post-implant. Of these, 13 deaths were attributed to infection, predominantly prior to engraftment [123]. Another center that performs this procedure performed a thymic implant on two patients with homozygous *PAX1* deficiency and congenital athymia. HLA matching is not required in this procedure, although the host should be assessed for HLA antibodies, and if present, thymic tissue matching those antibodies should not be used. Overall, this procedure, pioneered and led by M. Louise Markert, MD, PhD, is a treatment proven to enhance survival in a condition that is almost otherwise uniformly fatal by age 3 years. The pre- and peri-implant period should include PJP prophylaxis (initiated at 1 month of age), antifungal prophylaxis, IGRT, and azithromycin in these patients. Palivizumab should also be considered for patients with congenital athymia in areas where respiratory syncytial virus is circulating.

Relatively recently, the term thymic transplant has been replaced by thymic implant. Thymic implant involves harvesting infant donor thymus and incubating the tissue for approximately 2 weeks to ensure elimination of mature T cells which if present might lead to GVHD [2]. Thin slices of the harvested thymus are then implanted into the quadriceps muscle. Host stem cells make their way to the implanted tissue and as early as 3 to 4 months emerge as functional naïve T cells [2]. T cell production, while not attaining normal levels, reaches sufficient numbers to provide adequate and sustained immune function to prevent serious or opportunistic infections. In clinical trials of cultured thymic tissue implantation, biopsies demonstrated Hassall’s bodies in approximately 80% of patients [34], and circulating naïve T cells were detectable by 6 months post-implantation [124, 125]. Immune reconstitution significant enough to provide protection against infections generally occurred between 6 and 12 months but as late as 2 years post-implant [13]. In 2021 in the USA, thymic implantation was FDA approved as a service through a private corporation. This procedure is also performed in the UK [126].

## 22q11.2del with Congenital Athymia and Autologous Immune Dysregulation

22q11.2del with congenital athymia can result in a severe form of immune dysregulation due to autologous immune dysregulation (Omenn-like syndrome) [34]. This complication does not typically occur at birth but often manifests within the first few months of life when a small number of autoreactive and oligoclonal T cells peripherally expand. Signs and symptoms can manifest before a thymic implant is performed. In a cohort of 89 patients diagnosed with congenital athymia consented for thymus transplantation at one center, 43 (48%) met the criteria for this form of autologous immune dysregulation [35, 125]. T cell infiltration into various tissues and organs can result in organ damage. Cutaneous manifestations include an erythematous and eczematous-like dermatitis secondary to T cell infiltration of the dermis. Lymph node histology is consistent with dermatopathic lymphadenitis [13]. Infiltrates involving the liver may result in a transaminitis [124]. Gut involvement can lead to persistent diarrhea and failure to thrive [13, 99]. The associated Th2 skewing manifests as severe erythroderma, eosinophilia, and elevated IgE levels [127]. Due to this extrathymic oligoclonal expansion, not all affected patients have a persistent T cell lymphopenia [13, 34]. Flow cytometry of circulating T cells shows a predominance of memory T cells (CD45RO^+^ CD4^+^) [2]. Of note, such individuals may demonstrate proliferation to PHA despite having congenital athymia [95]. A disproportionately high number of double-negative T cells (DNTCs) with evidence of activation through assessment of CD25 and HLA-DR may also be detected [13, 95]. Additionally, the TCR Vß repertoire typically is severely restricted. Because these cells develop as a result of peripheral expansion of a very small number of oligoclonal T cell bands, they provide little to no immunity from infection [2, 34]. Management of this condition typically requires the use of immunosuppressive agents, which may include cyclosporin, tacrolimus, anti-thymocyte globulin, steroids, or alemtuzumab (anti-CD52). Cytotoxic T cell infusions can be considered if the patient has evidence of active infection. SCID and other causes of DTD should be ruled out. Transplacental maternal engraftment as the underlying cause of similarly presenting symptoms should also be assessed. This can be done through chimeric studies confirm circulating T cells are host rather than maternally derived circulating T cells [95] and evaluation using short-tandem repeat analysis, which is more sensitive and can be utilized in both males and females.

## Hematopoietic Stem Cell Transplant (HSCT)

Survival in patients with congenital athymia who undergo HSCT is only 41%, with a GVHD incidence of 50% [128]. A favorable outcome of hematopoietic stem cell transplant for athymia due to 22q11.2del is more likely when an HLA-matched sibling donor is available [129]. However, because of the persistent failure of thymopoiesis, maturation and regeneration of naïve T cells remain greatly hindered, as T cell development is generally limited to peripheral expansion [128]. Thus, HSCT is not recommended when a thymic implant remains an option, but a matched HSCT may represent an option in resource constrained settings.

## Immune Dysregulation and Autoimmunity

The thymus plays a fundamental role in establishing and maintaining central and peripheral immune tolerance [130]. T cell disorders are often associated with autoimmune disease, and individuals with 22q11.2del are at increased risk for autoimmunity [89, 93, 96, 131, 132]. The overall incidence of autoimmune disease is approximately 10% [96]. Manifestations include idiopathic thrombocytopenia (ITP) (4%), juvenile idiopathic arthritis (2%), thyroid disorders, autoimmune hemolytic anemia, and autoimmune enteropathies including celiac disease [133]. Numerous explanations have been postulated to explain the immune dysregulation associated with 22q11.2del. They include smaller thymic size, perturbed thymic architecture, defects in thymocyte development, and a decrease in regulatory T cell (Treg) production [66, 134, 135]. These irregularities can lead to an impaired central tolerance regarding positive and negative T cell selection and consequential escape of autoreactive thymocytes resulting in immune dysregulation and autoimmunity [66]. Tregs originate in the thymus, and some research shows that affected individuals may develop insufficient production of Tregs with age [131], as the Foxp3 levels that control CD4^+^CD25^+^ Tregs decrease after age 6 years [131]. Autoreactive T cells may also be less likely to be deleted in 22q11.2del due to thymic dysregulation and reduced induction of apoptosis and autoreactive T cells [104]. Reduced naïve CD4 T cell populations have been associated with an increased incidence of autoimmunity in patients [136]. Very low T cell counts and more rapid conversion from naïve to memory T cells have been postulated as predictors of immune cytopenia [137]*.* Autoimmune thyroid disease (autoantibody positive, Hashimoto thyroiditis, or Graves’ disease) was also associated with lower CD4 counts [132]. IgA deficiency, known to be associated with autoimmunity [138, 139], is also more prevalent in 22q11.2del.

## Atopy in 22q11.2del

Homeostatic proliferation in mice is associated with Th2 skewing [140, 141]. More recently, this phenomenon has also been demonstrated in humans with 22q11.2del, where a Th1 phenotype early in life evolved toward a Th2 cytokine profile phenotype in adults when compared to controls [99]. Individuals with 22q11.2del were found to have an increased incidence of both eczema and asthma but not allergic rhinitis [142].

## Conclusion

The work of many authors and their publications over many years have addressed immunological aspects of 22q11.2del/DGS. Despite this, widely variable approaches of initial and long-term assessment as well as how to best manage affected individuals have continued. By forming an expert workgroup to discuss how to best diagnose and care for affected individuals, and incorporating pertinent information gained from previously published literature, the hope is that these guidelines will provide clinicians caring for these patients with an updated clinical practice framework that is both comprehensive and practical as it relates to immunology.

## Data Availability

Data was obtained from journal articles cited and through discussions among the expert panel workgroup.

## Abbreviations

22q11.2del: 22Q11.2 deletion syndrome
AAAAI: American Academy of Allergy Asthma and Immunology
BCG vaccine: Bacillus Calmette–Guerin vaccine
CCR7: C-chemokine receptor 7
CD31: Cluster of differentiation 31
CD62L: Cluster of differentiation 62 L-selectin
CHARGE syndrome: Coloboma, heart defect, atresia choanae, retardation of growth and development, genital hypoplasia, ear anomalies/deafness (28)
CHD7: Chromodomain helicase DNA-binding protein 7
CID: Combined immune deficiency
CMA: Chromosomal microarray
cTEC: Cortical thymic epithelial cells
DGS: DiGeorge Syndrome
DNA-PKcs: DNA-dependent protein kinase catalytic subunit
DTD: Defect in thymic development
ESID: European Society for Immunodeficiencies
FOXI3: Forkhead box I3
FOXN1: Forkhead box N1
GVHD: Graft-versus-host disease
IGRT: IgG replacement therapy
MAC: *Mycobacterium avium* Complex
mTEC: Medullary thymic epithelial cells
NBS: Newborn screen
OFCS2: Otofaciocervical syndrome type 2
PAX1: Paired box 1
PJP: *Pneumocystis jirovecii* Pneumonia
RTE: Recent thymic emigrant
SAD: Specific antibody deficiency
SCID: Severe combined immune deficiency
TB: Tuberculosis
TBNK: T cell, B cell, and Natural killer cell
TBX-1: T box transcription factor 1
TCL: T cell lymphopenia
TCR Vβ: T cell receptor V-beta repertoire
TEMRA: T effector memory re-expressing RA
TRECs: T cell receptor excision circles
TREGs: T regulatory cells
USIDNET: US Immunodeficiency Network
VCFS: Velocardiofacial syndrome
