# Correction to: Clinical Practice Guidelines for the Immunological Management of Chromosome 22q11.2 Deletion Syndrome and Other Defects in Thymic Development

**DOI:** 10.1007/s10875-024-01655-3

**Published:** 2024-01-22

**Authors:** Peter J. Mustillo, Kathleen E. Sullivan, Ivan K. Chinn, Luigi D. Notarangelo, Elie Haddad, E. Graham Davies, Maria Teresa de la Morena, Nicholas Hartog, Joyce E. Yu, Vivian P. Hernandez-Trujillo, Winnie Ip, Jose Franco, Eleonora Gambineri, Scott E. Hickey, Elizabeth Varga, M. Louise Markert

**Affiliations:** 1 Division of Allergy and Immunology, Department of Pediatrics, Nationwide Children’s Hospital, Columbus, OH 43205 USA; 2 Division of Allergy Immunology, Department of Pediatrics, Children’s Hospital of Philadelphia, Philadelphia, PA 19104 USA; 3 Division of Immunology, Allergy, and Retrovirology, Department of Pediatrics, Texas Children’s Hospital, Houston, TX 77030 USA; 4 National Institute of Allergy and Infectious Diseases, National Institutes of Health, Bethesda, MD 20892 USA; 5 Department of Pediatrics, Department of Microbiology, Infectious Diseases and Immunology, CHU Sainte-Justine, University of Montreal, Montreal, QC H3T 1C5 Canada; 6 Department of Immunology, Great Ormond Street Hospital and UCL Great Ormond Street Institute of Child Health, London, WC1N 3HJ UK; 7 Division of Immunology, Department of Pediatrics, Seattle Children’s Hospital, University of Washington, Seattle, WA 98105 USA; 8 Spectrum Health Helen DeVos Children’s Hospital Department of Allergy and Immunology, Michigan State University College of Human Medicine, East Lansing, USA; 9 Division of Allergy, Immunology & Rheumatology, Department of Pediatrics, Columbia University Irving Medical Center, New York, NY USA; 10 Division of Allergy and Immunology, Department of Pediatrics, Nicklaus Children’s Hospital, Miami, FL USA; 11 Department of Immunology, Great Ormond Street Hospital and UCL Great Ormond Street Institute of Child Health, London, WC1N 3JH UK; 12 Grupo de Inmunodeficiencias Primarias, Facultad de Medicina, Universidad de Antioquia UdeA, Medellin, Colombia; 13 Department of “NEUROFARBA”, Section of Child’s Health, University of Florence, Florence, Italy; 14 Centre of Excellence, Division of Pediatric Oncology/Hematology, Meyer Children’s Hospital IRCCS, Florence, Italy; 15 Division of Genetic & Genomic Medicine, Department of Pediatrics, Nationwide Children’s Hospital, Columbus, OH 43205 USA; 16 Institute for Genomic Medicine, Nationwide Children’s Hospital, Columbus, OH 43205 USA; 17 Department of Immunology, Duke University Medical Center, Durham, NC 27710 USA

**Correction to: Journal of Clinical Immunology**

10.1007/s10875-022-01418-y

The institutional affiliation #14 for Eleonora Gambineri was incorrectly listed on the first page of the original article. Eleonora Gambineri’s correct affiliation #14 is Centre of Excellence, Division of Pediatric Oncology/Hematology, Meyer Children’s Hospital IRCCS, Florence, Italy.

The original version has been corrected.

